# Documentation of older people’s end-of-life care in the context of specialised palliative care: a retrospective review of patient records

**DOI:** 10.1186/s12904-021-00771-w

**Published:** 2021-06-24

**Authors:** M. Sjöberg, A.-K. Edberg, B. H. Rasmussen, I. Beck

**Affiliations:** 1 Department of Care Science, Faculty of Health and Society, Malmö University, Malmö, Sweden; 2 The Research Platform for Collaboration for Health, Faculty of Health Sciences, Kristianstad University, Kristianstad, Sweden; 3 The Institute for Palliative Care, Lund University and Region Skane, Lund, Sweden; 4 Department of Health Sciences, Faculty of Medicine, Lund University, Lund, Sweden; 5 Department of Clinical Sciences, Faculty of Medicine, Lund University, Lund, Sweden

**Keywords:** Palliative care, Elderly, Retrospective review, Documentation, Patient records

## Abstract

**Background:**

Palliative care focuses on identifying, from a holistic perspective, the needs of those experiencing problems associated with life-threatening illnesses. As older people approach the end of their lives, they can experience a complex series of problems that health-care professionals must identify and document in their patients’ records. Documentation is thus important for ensuring high-quality patient care. Previous studies of documentation in older people’s patient records performed in various care contexts have shown that such documentation almost exclusively concerns physical problems. This study explores, in the context of Swedish specialised palliative care, the content of documentation in older people’s patient records, focusing on documented problems, wishes, aspects of wellbeing, use of assessment tools, interventions, and documentation associated with the person’s death.

**Methods:**

A retrospective review based on randomly selected records (*n* = 92) of older people receiving specialised palliative care, at home or in a palliative in-patient ward, who died in 2017. A review template was developed based on the literature and on a review of sampled records of patients who died the preceding year. The template was checked for inter-rater agreement and used to code all clinical notes in the patients’ records. Data were processed using descriptive statistics.

**Results:**

The most common clinical notes in older people’s patient records concerned interventions (*n* = 16,031, 71%), mostly related to pharmacological interventions (*n* = 4318, 27%). The second most common clinical notes concerned problems (*n* = 2804, 12%), pain being the most frequent, followed by circulatory, nutrition, and anxiety problems. Clinical notes concerning people’s wishes and wellbeing-related details were documented, but not frequently. Symptom assessment tools, except for pain assessments, were rarely used. More people who received care in palliative in-patient wards died alone than did people who received care in their own homes.

**Conclusions:**

Identifying and documenting the complexity of problems in a more structured and planned way could be a method for implementing a more holistic approach to end-of-life care. Using patient-reported outcome measures capturing more than one symptom or problem, and a systematic documentation structure would help in identifying unmet needs and developing holistic documentation of end-of-life care.

**Supplementary Information:**

The online version contains supplementary material available at 10.1186/s12904-021-00771-w.

## Background

The documentation included in patient records should capture the care provided and ensure that continuous high-quality care is given across the physical, psychological, social, and existential dimensions [1, 2]. Clinical record-keeping is integral to good professional practice and the delivery of high-quality health care [3]. More specifically, good clinical record-keeping enables continuity of care and improves the communication of information, which is important for evaluating performed interventions and planning future care [2]. Appropriate documentation implies adherence to a plan for providing care and evaluating performed interventions; this should include consideration not only of patients’ problems but also of patients’ beliefs and values [4]. Previous studies show that hospital- and nursing-home-managed records of older people at the end of their lives frequently include documentation of physical problems, but rarely note psychological, social, or existential problems [5, 6]. Palliative care has a pronounced emphasis on holistic care [7], so we can expect ample attention to psychological, social, and existential problems, beliefs, and values in the documentation in older people’s patient records in specialised palliative care.

As multimorbidity increases with age [8], end-of-life care for older people demands specific attention to the complexity of multiple symptoms and needs. Problems such as pain, dyspnoea, sleeplessness, fatigue, and feeding problems are common among older people near the end of their lives [9, 10]. A study of a group of older people with end-stage heart failure observed a total of 21 different problems during their final months of life, with an average of seven problems per person, the most common being breathlessness, pain, fatigue, and anxiety [11]. Older people at the end of life have unmet symptoms and needs, such as pain, weakness, emotional distress, and anxiety to the same levels as people with cancer, and a greater need of information compared to younger people [12, 13]. Problems such as pain have been shown to have several dimensions, such as physiological, behavioural, sensorial, affective, cognitive, sociocultural, and spiritual dimensions [14]. Furthermore, multimorbidity is highly prevalent among older people and is estimated to exceed 80% for those aged ≥85 years [8], leading to various interrelated symptoms. To capture older people’s complex problems and needs, systematic assessments of symptoms and risks would seem to be crucial. For patients with complex problems, systematic assessments have been shown to be effective for recognising symptoms, other than pain, that are affecting the person [15–17]. Recommendations from the European Association of Palliative Care (EAPC) highlight the importance of using multidimensional measures covering various symptoms related to physical, psychological, social, and spiritual domains. It is further of importance to continuously evaluate baseline assessments [15]. Patient-centred outcome measures have also been shown to be suitable for improving emotional and psychological patient outcomes, and for promoting conversation regarding quality of life [18], emphasising the importance of communication between patients and health-care professionals (HCPs) to create awareness of patients’ unmet problems.

Body, mind, and spirit interact and influence wellbeing and illness [19]. The mind is integrated with the body and, in a palliative care context, it has been shown that patients’ symptoms remind them of their impending deaths [20]. A situation of increasingly losing connection with the surrounding world and not being taken seriously by others, is shown to increase the risk of older people wishing to end their life [21]. Studies have indicated that older people generally wish to talk about their lives, dying, and death [22], but also sometimes do not want to think about death, acceptance of death, or the longing for relief [23]. It is reasonable to assume that it is crucial to pay attention to and document the problems older people experience.

A previous study of end-of-life patients in nursing homes that cared for older people with dementia revealed that dialogue with patients regarding their care, wishes, and death was not included in the associated documentation [6]. This finding accords with the results of interviews with HCPs who cared for older persons in home care, nursing home care, palliative care, primary care, hospital care, and pre-hospital care in Sweden [24], revealing that these HCPs, when caring for frail older persons, experienced insecurity and fear of discussing existential issues. Palliative care particularly focuses on identifying, using a holistic approach, the needs of people experiencing life-threatening illnesses [25] and on providing complete care by addressing physical, psychological, social, and existential pain [26] at end of life. In line with this, a study by Sundström and co-workers found that HCPs working in specialised palliative care could more easily address existential concerns than could HCPs working in primary care, nursing homes, or hospitals [27]. However, we have limited knowledge of the extent to which HCPs in specialised palliative care grasp the complexity of older people’s problems and needs, and of the extent to which this complexity is observed, assessed, documented, and evaluated.

### Aim

The aim of this study was to explore, in the context of Swedish specialised palliative care, the content of documentation in older people’s patient records.

Research questions:

What problems, wishes, aspects of wellbeing, assessment tools, and interventions are documented in patient records and to what extent? What is documented in association with the patients’ deaths and to what extent?

## Methods

### Design

This study constitutes a retrospective review based on the records of 92 older patients receiving specialised palliative care. A review template was developed and used to code all clinical notes in the patients’ records; the resulting coding was processed using descriptive statistics.

### Context and setting

In this study, we examined patient records from 11 units of a specialised palliative care clinic in three geographical areas located in southern Sweden. This specialised palliative care approach uses teams having expert competence in palliative care to address the complex needs of patients and their families; the patients are of all ages, with life-threatening or debilitating chronic illnesses such as cancer, lung and heart diseases, neurological diseases, and multimorbidity. The clinic can care for approximately 280 patients at home and approximately 70 patients in palliative in-patient wards. Depending on their needs, patients can transfer between home and the wards at any time during the care. About 1650 patients die at the clinic annually, of whom approximately 54% are aged ≥75 years [28]. In total, the clinic has approximately 450 employees: physicians (8%), registered nurses (60%), and other professionals (32%). The interdisciplinary care teams are led by the physicians and comprise registered nurses, licensed practical nurses, physiotherapists, occupational therapists, dieticians, and social workers. The last four types of professionals work during the daytime, while the physicians, registered nurses, and licensed practical nurses are available 24 h a day. Licensed practical nurses work only in the in-patient wards.

In Sweden, the Patient Data Act [29] states that all licensed HCPs are obliged to maintain records for all patients, and that this documentation should be available for the patients to read. The Swedish Register of Palliative Care (a national quality register for care provision during the last week of life) includes a field for registering whether an end-of-life conversation was held with the patient [28]. This conversation should be held with the patient and, if applicable, his/her relative(s) at the time of his/her transition from curative to palliative care and should include information and planning regarding the dying process [30].

### Sample

The sample comprised patients’ records sourced from the 11 units of the palliative care clinic for people aged ≥75 years who had died between 1 January and 31 December 2017 and who had been cared for in an in-patient ward and/or in the home. The ≥75-year age cut-off was chosen based on criticisms of defining older people as those having a chronological age of ≥65 years, as those under 75 years old are generally still robust and active with well-maintained mental and physical health (e.g. [31]). Data were obtained from electronic patient records. Of the total number of records (*n* = approximately 750) of deceased older people in 2017, one of every eight records was randomly selected in each of the three geographical areas, for a total of 92 records included in this study.

### Data collection

**Fig. 1:**
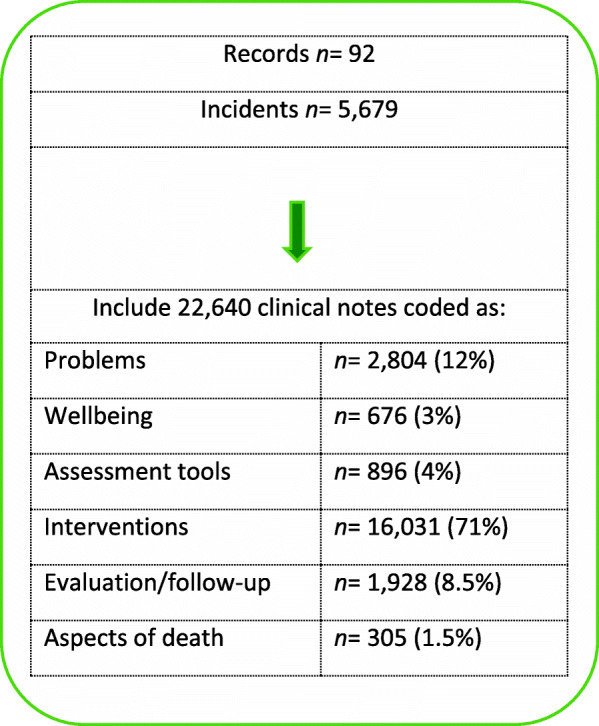
Overview of documented incidents and clinical notes

Using the review template described below, the documentation during the first and last months of care for each record was reviewed. For records with documentation exceeding these two months (*n* = 23), the documentation for the additional time was read in case new information not previously documented was found; new information was found in 16 records, and this was included in the data material. Overall, in the 92 reviewed patients’ records, 5679 incidents (i.e., occasions when the HCP met the patient) were identified, and 22,640 clinical notes (i.e., notes made by HCPs) were coded (Fig. 1). Of the clinical notes, 81.6% were made by registered nurses, 13.4% by physicians, and 5% by other professionals.

### Development of a review template

The review template was developed in several steps. First, the literature concerning (a) symptoms and other concerns commonly occurring at end of life [32] and (b) psychosocial and existential aspects important to older people at end of life [33–35] was reviewed. This review resulted in a list of symptoms and other aspects important for older people at end of life. Second, a content analysis of 16 records of patients who died in 2016 at the included study units was performed (these records were not included in the study sample) and the abovementioned list was complemented with additional aspects. Finally, categories and subcategories were identified from the list and the review template was structured accordingly.

The review template was pilot tested using a sample of another 16 records of patients who died in 2017 and was revised by the first and last authors until it was considered functional. The pilot-tested records were later included in the study. The final template comprised two parts: the first covered demographics; the second part contained two levels of abstractions with 33 categories and 264 subcategories, see Table 1. An example of the category “Body and skin care”, together with the subcategories and their codes, is provided in Table 2. The content of the subcategories reflects the clinical notes on a concrete level. All clinical notes were coded with the category and subcategory numbers and entered into IBM SPSS Statistics, version 25 [36].

**Table 1:** Categories and number of subcategories included in the review template

	Categories	Number of sub-categories
1	Diagnosis	8
2	Place of care	3
3	Patient’s wishes and priorities, i.e., notes about the patient’s wishes or about aspects of importance	5
4	Strategies, i.e., notes about how the patient handles her/his situation	2
5	Wellbeing, i.e., notes about the patient’s strengths and sense of emotional balance, meaning, and community	8
6	Problems, symptoms, and needs	45
7	Documented by whom	7
8	Notes about intervention(s) in relation to the identified problem	2
9	Notes about evaluation in relation to the identified problem	2
10	Functional status	4
11	Assessment tool used	11
12	Documented by whom	7
13	Notes about the outcome score of the assessment	2
14	Interventions based on the outcome score of the assessment	7
15	Care plan generated based on the assessment	3
	Interventions:	
16	Activity/mobilisation	8
17	Body and skin care	10
18	Communication/conversation	10
19	Elimination	14
20	Environmental adaptations	5
21	Nutrition	12
22	Planning, coordination, and collaboration	18
23	Presence and touch	5
24	Respiration and circulation	16
25	Pharmacological	12
26	Type of drug	15
27	Intervention documented by whom	7
28	Relatives’ wishes and priorities, i.e., notes about relatives’ wishes or about aspects of importance	2
29	Note written during the patient’s last week of life	2
30	Relatives present during the patient’s last days of life	2
31	People present at the moment of the patient’s death	4
32	Place of death	4
33	Bereavement follow-up	2

**Table 2:** One of the 33 categories with subcategories included in the review template

Category 17: Body and skin care	
**Subcategories**	**Codes**
Assistance with washing/showering	1
Assistance with dressing/undressing	2
Assistance with hair care	3
Assistance with manicure/pedicure	4
Assistance with shaving	5
Information about self-care	6
Inspection of skin	7
Soothe and calm skin	8
Lubricate dry skin	9
Wound dressing	10

### Audit of the patient records

The first author reviewed the records, identified the clinical notes, coded them in accordance with the review template, and entered the data into an SPSS file. To ensure the reliability of the selected relevant segments of content from the records, as well as the coding into relevant subcategories, a selection of the records was also reviewed by two of the co-authors (I.B. and B.R.). Of the 5679 documented incidents, 278 (5% of the total) were checked for inter-rater agreement at various points during the data collection. Every documented incident was tagged with several codes; 1159 codes were included in the inter-rater-agreement analysis, and agreement was reached for 1125 of them (97%). The codes not agreed on were discussed until consensus was reached, and these codes were then adjusted accordingly in the already reviewed records.

### Analysis

All statistical analyses were performed using IBM SPSS Statistics, version 25 [36]. Descriptive data were summarised using frequencies, percentages, and, when possible, in terms of means, medians, standard deviations, and ranges. The statistics were calculated using the Chi-square test when comparing groups and Fisher’s exact test when the expected cell count was less than 5 [37].

## Results

The results are based on the coded content of notes in the records of 92 deceased older people, 36 (39%) women and 56 (61%) men. Thirty-four per cent of the men and 58% of the women (*p* = 0.049) lived alone. Thirty-two per cent died in their homes and 68% in in-patient wards (Table 3). Twenty-two per cent of those living alone and 38% of those cohabiting died at home. The time between enrolment and death ranged from 2 to 312 days, with a mean of 55 days (median 20 days); for those who received care at an in-patient ward only, the range was 2–118 days, with a mean of 16 days (median 9 days), and for those who received care at home only, the range was 2–247 days, with a mean of 75 days (median 47 days). The most common documented diagnosis for enrolment was cancer (81%, *n* = 74), while 19% (*n* = 18) had other diagnoses. There were significant gender differences in this regard, and a larger share of women (92%, *n* = 33) than of men (72%, *n* = 41) (*p* = 0.024) had cancer as the documented reason for enrolment (Table 3). Enrolment visits, performed either at the hospital or in the older people’s own homes, were usually made jointly by a physician and a registered nurse (98%, *n* = 90). Information regarding the older people’s relatives, most of whom were spouses (53%, *n* = 49) and children (35%, *n* = 32), was present in all patients’ records.

**Table 3:** Demographic data of the sample, *n* = 92

Demographics	***n*** (%)	Women *n* = 36 (39%)	Men *n* = 56 (61%)	***p-value***
Age, mean (SD) Range	82.3 (4.5) 75–94 years	82.7 (5.4) 75–94 years	82 (3.9) 75–92 years	
		***n*** **(%)**	***n*** **(%)**	
Living alone	40 (43.5)	21 (58)	19 (34)	***0.049***^***2***^
Living together	52 (56. 5)	15 (42)	37 (66)	
Relatives				*0.171*^*2*^
Spouse	49 (53)	15 (42)	34 (61)	
Children	32 (35)	15 (42)	17 (30)	
Other	10 (11)	6 (16)	4 (7)	
Nobody	1 (1)	–	1 (2)	
Diagnosis^1^				***0.029***^***3***^
Cancer	74 (81)	33 (92)	41 (73)	
Lung disease (other than cancer)	4 (4)	1 (3)	3 (5)	
Neurological disease (ALS, Parkinson’s)	3 (3)	**–**	3 (5)	
Heart disease	2 (2)	**–**	2 (4)	
Two or more diagnoses	9 (10)	2 (5)	7 (13)	
Place of care				*0.182*^*2*^
Only palliative in-patient ward	39 (42.5)	11 (30.5)	28 (50)	
Only ordinary home	25 (27)	12 (33.5)	13 (23)	
Combination palliative inpatient ward/ ordinary home	28 (30.5)	13 (36)	15 (27)	
Place of death				*0.495*^*3*^
Palliative inpatient ward	63 (68)	23 (64)	40 (71.5)	
Ordinary home	29 (32)	13 (36)	16 (28.5)	

^1^ Diagnosis related to enrolment in the palliative care unit, ^2^ Fisher’s exact test, ^3^ Chi-square test

### Documented problems

The numbers of documented problems ranged between 1 and 15 problems/record (*N* = 92), and in 81% (*n* = 75) of the records more than six problems were documented. Pain was the most commonly documented problem (30%, *n* = 848 clinical notes), followed by respiratory and circulatory problems (19%, *n* = 529 clinical notes) and anxiety (10%, *n* = 278 clinical notes) (Table 4). In 115 (14%) clinical notes, pain was documented in clusters; specifically, in combination with anxiety in 96 clinical notes, in combination with nausea in 16 clinical notes, and in combination with both nausea and anxiety in three clinical notes. Some problems were documented to a lesser extent during the last week of the patients’ lives. For example, nutrition and fatigue were documented in 72% (*n* = 314) and 78% (*n* = 103) of clinical notes in the records, respectively, before the last week of life, but in just 28% (*n* = 124) and 22% (*n* = 30) of clinical notes, respectively, during the last week of life (Table 4). Clinical notes regarding anxiety and a general worsening condition became more common during the last week of life (Table 4).

**Table 4:** Clinical notes documenting problems (*n* = 2804) and wishes and wellbeing (*n* = 676)

Area	All notes ***n*** (%)	Before last week^**1**^ ***n*** (%)	Last week^**2**^ ***n*** (%)
Problems			
Pain	848 (30)	479 (56)	369 (44)
Respiratory/bleeding/circulation	529 (19)	302 (57)	227 (43)
Nutrition problems	439 (16)	314 (72)	124 (28)
Anxiety	278 (10)	109 (37)	169 (63)
Fatigue	133 (5)	103 (78)	30 (22)
Sleep problems	91 (3)	66 (73)	25 (27)
Delirium	77 (3)	42 (54)	35 (46)
Mood	69 (2)	58 (84)	11 (16)
Depressed/sad	53		
Angry/frustrated/dissatisfied	16		
Fall/fall tendency	53 (2)	46 (87)	7 (13)
Skin problems	50 (2)	33 (67)	17 (33)
Movement difficulties	42 (2)	31 (74)	11 (26)
Neurological problems	13 (< 1)	5 (38)	8 (62)
Worsened general condition/death	182 (6)	48 (27)	133 (73)
Wishes and wellbeing	676	549 (81)	127 (19)

^1^ Documented before last week of life (*n* = 71 records), ^2^ Documented during the last week of life (*n* = 92 records)

### Documented wishes and aspects of wellbeing

The older people’s wishes and/or aspects of their wellbeing were documented in 62 (67%) records, corresponding to a total of 676 clinical notes (Table 5). Of these, 205 (31%) clinical notes documented wishes expressed by the older people themselves (Table 5). These wishes related to care, treatment, the care setting, and, in a few cases, where they would like to die. The patients also expressed a desire to be more active and independent, and to have a conversation with a priest, social worker, or physician; in contrast, in three cases it was emphasised that the patient did not want to talk about death or dying at all. Aspects of wellbeing, such as having enough energy to participate in activities, socialising, and strategies for managing the situation, were documented in 471 (69%) clinical notes; of these, the person’s strategies for managing the situation were documented in 3% (*n* = 18) of the clinical notes.

**Table 5:** Clinical notes documenting wishes and wellbeing (*n* = 676)

Contents of the clinical notes	***n*** (%)
Wishes important to the patient	205 (31)
Laughs/is in a good mood/ wellbeing	128 (19)
Sleeps well	105 (15)
Socialises with relatives/friends	90 (13)
Engages in activities	71 (10)
Has good help from relatives	41 (6)
Looks forward to something	18 (3)
Has strategies for handling the situation	18 (3)

### Documented use of assessment tools

Use of various standardized assessment tools was documented in almost every record (*n* = 89) in a total of 895 clinical notes; the most common type was self-assessed symptom-assessment tools, mentioned in 564 clinical notes (63%). Of these, the most common was a single-symptom assessment tool measuring pain, both at enrolment and during the care period. Risk assessment tools to identify risk of fall, pressure sores and oral health problems were used both at enrolment and at regular intervals during the care period (Table 6). For an overview of the use of standardized assessment tools and, the symptoms and risks that were assessed, see Table 6 and Additional file 1.

**Table 6:** Documented use of assessment tools (*n* = 895) in 89 patient records

Type of assessment	Symptoms, concerns or risks included in the tool	Assessment tool	Used			
			during first week of care^**1**^ *n* = 142 (16%)	during care period^**1**^ *n* = 215 24%)	in case of a problem *n* = 345 (38%)	to follow up previously documented problem *n* = 194 (22%)
**Symptom assessment**	Multiple symptoms; 10 symptoms^2,a^,	Edmonton Symptom Assessment System, ESAS [38]	5 (4)	8 (4)	–	1 (< 1)
	Multiple symptoms and concerns;14 symptoms and 3 concerns^2,b,^	Integrated Patient care Outcome Scale, IPOS [39]	3 (2)	18 (8)	4 (1)	7 (4)
	Two symptoms; anxiety and depression^2^	Hospital Anxiety and Depression Scale, HADS [40]	2 (1)	1 (< 1)	–	–
	Single symptom e.g. pain^2^	Numerical Rating Scale, NRS [41]; Verbal Descriptive Scale, VDS [41]; Visual Analog Scale, VAS [41]	14 (10)	41 (19)	297 (86)	164 (84)
	Single symptom; pain^3^	Abbey Pain Scale [42]; Face, Legs, Activity, Cry, Consolability scale, FLACC [43]	2 (1)	3 (1)	41 (12)	20 (10)
	**Total**		25 (18)	71 (33)	342 (99)	192 (99)
**Risk assessment**	Single risk; fall^3^	Downton Fall Risk Index, DFRI [44]	36 (26)	23 (11)	1 (< 1)	–
	Single risk; pressure sore^3^	Norton Pressure Sore Risk-Assessment Scale, Norton Scale [45]	33 (23)	24 (11)	1 (< 1)	–
	Single risk; oral health^3^	Revised Oral Assessment Guide, ROAG [46]	47 (33)	97 (45)	1 (< 1)	2 (1)
	**Total**		116 (82)	144 (67)	3 (1)	2 (1)

^1^ Used without any problem being documented

^2^ Self assessment

^3^ Observation

^a^ pain, shortness of breath, fatigue, nausea, appetite, drowsiness, anxiety, depression, sleep, feeling of wellbeing

^b^ pain, shortness of breath, weakness or lack of energy, nausea, vomiting, poor appetite, constipation, sore or dry mouth, drowsiness, poor mobility, anxiety, family anxiety, feeling at peace, sharing feelings, information, practical matters

All documented care plans were generated based on the outcome of standardized risk-assessment tools: for preventing and treating oral problems in 95 cases; for pressure sores prevention in 44 cases; and for avoiding fall accidents in 39 cases.

### Documented interventions

Overall, the records (*N* = 92) containing clinical notes regarding the interventions performed to address problems featured a total of 16,049 clinical notes. Of the clinical notes documenting interventions, 4318 (27%) were related to pharmacological interventions. Most of the pharmacological interventions involved treatment of pain (18%, *n* = 777), followed by treatment of breathing problems (7%, *n* = 302) and anxiety (5%, *n* = 216). Of the total clinical notes documenting interventions, 891 (6%) concerned conversations, of which 580 (65%) were support related to a problem and the remaining 35% (*n* = 312) involved active listening or support related to the patient’s life situation or experiences. Of these 580 clinical notes, 26% (*n* = 151) concerned conversation with the patient alone, 9% (*n* = 52) conversation with the patient and relative(s) together, and 30% (*n* = 174) conversation with the relative(s) alone. Other documented interventions concerned various problem areas, such as respiration and circulation (2252 clinical notes, 14%), elimination (1692, 10%), nutrition (1627, 10%), activity and mobilisation (1217, 7%), and skin and body care (776, 5%), whereas other documented interventions concerned creating peacefulness, for example, via tactile massages and closeness (774, 5%) and adaptation of the physical environment (37, < 1%).

### Documentation associated with the person’s death

In 86% (*n* = 79) of the records, one or more end-of-life conversations were documented. This conversation was held with the older person alone in 15% (*n* = 12) of cases, with the older person together with his/her relative(s) in 42% (*n* = 33) of cases, and with the relative(s) alone in 43% (*n* = 34) of cases. According to the clinical notes, the end-of-life conversation was conducted by physicians in 107 cases and by registered nurses in 18 cases. All records (*n* = 92) documented place of death. According to this documentation, 27% (*n* = 17) of those who died in the in-patient ward and 7% (*n* = 2) of those who died at home died without anyone present. The documentation also showed that HCPs were present for 26% (i.e., 16 of 63) of the deaths that occurred in the in-patient ward and for 38% (i.e., 11 of 29) of the deaths that occurred in the home (Table 7).

**Table 7:** Documentation of death: place, who was present, and follow-up conversation, *N* = 92

Place of death	***n*** (%)	Present at time of death	***n*** (%)
In-patient ward	63 (68)	Relative only	29 (46)
		Both relative and staff	10 (16)
		Staff only	6 (10)
		Nobody	17 (27)
		Not documented	1 (1)
At home	29 (32)	Relative only	14 (48)
		Both relative and staff	4 (14)
		Staff only	7 (24)
		Nobody	2 (7)
		Not documented	2 (7)
**Follow-up conversation with relative after patient’s death**	***n*** **(%)**		***n*** **(%)**
In-patient ward	63 (68)	Follow-up by telephone call	33 (36)
		Follow-up on the ward	4 (4)
		Follow-up by home visit	–
		Relative declined follow-up	5 (5)
		Not documented	21 (23)
Ordinary home	29 (32)	Follow-up by telephone call	20 (22)
		Follow-up on the ward	–
		Follow-up by home visit	2 (2)
		Relative declined follow-up	1 (1)
		Not documented	6 (7)

## Discussion

The aim of this study was to explore, in the context of Swedish specialised palliative care, the content of documentation in older people’s patient records. The study captures how care provision was documented and not how the actual care was provided; however, good clinical record-keeping is integral to good professional practice and the delivery of high-quality health care [3].

In summary, the main findings were that pain was the most documented problem. The most documented assessments were performed by using single symptom assessment tools, and were not followed up. The only assessments that generated care plans were risk assessments of fall, pressure sores and oral health problem. Further, it is well known that more favourable patient outcomes are achieved when baseline assessments are continuously followed up [15], increasing the likelihood of high-quality and safe care. Regarding documentation of dying and death, end-of-life conversations with the older person were only documented in approximately 60% of the records, and a higher percentage of older people died alone in the in-patient wards than in their own homes.

A model for person-centred documentation in the palliative care setting has been introduced and described by Ternestedt et al. [47]. This model is based on six keywords (i.e., self-image, symptom relief, self-determination, social relationships, synthesis, and strategies) that capture various dimensions and care needs and that characterise a death that could be considered good and meaningful for the individual. This tool could help HCPs with documentation and ensure that older persons’ wishes and needs are documented and paid appropriate attention. In addition, the model facilitates focussing the documentation on person-centred rather than problem-based aspects [47]. This accords with the guidelines of the British Geriatrics Society [48], which presents 11 principles of good death and dying, emphasising the persons’ own needs and preferences regarding the dying process. Examples of these principles are retaining control of what happens, having access to spiritual and emotional support, and having time to say goodbye. The use of a systematic documentation structure can help HCPs perform person-centred documentation and can also lead to the more holistic provision of care and documentation.

The results indicated that pain was the most documented problem and was mostly pharmacologically treated, in agreement with the findings of previous studies (e.g., 9, 10). In particular, Gunhardsson et al. [49] found that pain is documented more often than are other problems in specialised palliative care. Our results indicated that pain was mostly documented as a physical problem, suggesting that the psychological, social, and existential dimensions of pain (cf. 26) might not have been paid sufficient attention and may remain unknown to HCPs. Studies have shown the need to have a holistic understanding of pain – as captured in the concept of ‘total pain’ – as a prerequisite for pain control [50]. Symptoms rarely occur in isolation and feelings such as anxiety, fear, guilt, and anger have been shown to lower the pain threshold [51]. This highlights the need for the use of patient-reported outcome measures supporting the exploration of meanings and the understanding of experienced problems and symptoms [15]. Patient involvement is essential to capture the older patients’ own wishes and preferences [18] and to provide patients with opportunities to express their problems and needs [52], which, in turn, could be used to develop individual care plans.

There is a gap between the documented end-of-life care in the older people’s patient records and existing quality indicators of what constitutes a good death and dying. An end-of-life conversation with the older person was documented in only approximately 60% of the records. This is far below the target level of ≥98% recommended in the quality indicator of the Swedish National Board of Health and Welfare [53], which states that conversations with the patient regarding the content and direction of care at end of life are a high priority. Another quality indicator for palliative care is companionship at death [54]. The results of our study showed that 27% of the older people who died in the palliative in-patient wards died alone. This is higher than the figures found in previous studies, which report approximately 16% dying alone in specialised palliative care [55, 56]. The aim that nobody should die without companionship reflects the negative connotations of dying alone. A review to determine patients’, family members’, and HCPs’ definitions of what constitutes a good death reported that the relevant aspects concern where, when, and how death occurs [57]. The review presented dying in one’s sleep, dying free of pain, and dying after having addressed matters felt to be important as examples of good deaths. Although older people in nursing homes and family caregivers emphasise that having a human connection at the end of one’s life is important, some older people express a desire to die alone, while others are ambivalent but feel that it would not bother them if they died alone [58]. It is therefore important to document each person’s own wishes and needs, but also to ensure that there is ongoing discussion of these aspects, as it can be difficult to make predictive decisions regarding one’s own death.

Most (81%) of the older people covered in this study had a diagnosis of cancer, and only 19% had other diseases. This is in line with a German study of specialised palliative home care in which approximately 80% of the included patients had cancer and only approximately 20% had other diseases [59]. National Swedish statistics show that even though older people mostly die of circulatory and respiratory diseases, older people who receive care in specialised palliative care units predominantly have a cancer diagnosis [28]. How the end-of-life period manifests itself and affects people varies among individuals but is also influenced by the diseases and symptoms they have [60]. These symptoms must be carefully examined and diagnosed, which is crucial in order to assess the individual’s need for treatment. In acute stages, the focus is too often only on current symptoms that are treated with a drug without assessing the situation from a holistic perspective [61], possibly resulting in the older people’s problem being either under- or wrongly treated. Additionally, older people with non-cancer diagnoses have been found to have a higher symptom burden than do older people with cancer [9, 62], and even though they had severe palliative care needs, they gained access to palliative care later than did people with cancer [59]. Thus, there is a risk that older people with diseases other than cancer may have less access to specialised palliative care, even though their symptom burden and needs mean that they require such care. This is something that should be considered in future research.

### Strengths and limitations

The review template used here was developed and based on literature reviews and on reviewing the content of a sample of patient records; to verify that this template was valid, we conducted a pilot test of 16 records. The sample for this study was chosen at random from three geographical areas, which reduced the risk of selection bias [63] and increased the likelihood that the sample would be representative of the population of patient records for the entire region. Our sample is also representative of patients receiving specialised palliative care in the region, about 80% of whom are patients with cancer diagnoses [28]. To decrease the risk of observer bias on the part of the person performing the record review, an inter-rater reliability assessment was conducted. To ensure equivalent coding of the content of the records, two of the co-authors continuously audited the coded records, which reduced the risk of gradual change in how the records were coded [63]. A strength of the present research approach was that the registered nurses, physicians, and other HCPs at the included units all provided their documentation in the same records, giving a comprehensive picture of the documentation. However, the generalisability of the results could be influenced by the fact that the organisation and documentation of palliative care may differ between regions and countries.

## Conclusion

There is a need to consider and understand the complexity and multi-dimensionality of older people’s pain-related problems at end of life. The documentation showed that while pain and risk assessment tools were often used, other assessment tools were used only rarely. To address the complexity of needs among older people at end of life, patient documentation must be structured and tailored to comprehensively capture their needs. The documentation must be based on the people’s own needs and desires regarding the dying process, which should be determined through ongoing conversations.

To provide holistic end-of-life care, the task should be considered in terms of the physical, psychological, social, and existential aspects of suffering. This study indicates a need for HCPs to document older people’s palliative care needs in a more planned and structured manner. Use of patient-reported outcome measures capturing more than one symptom or problem could be one method of identifying, managing, and documenting unmet needs, if followed up by conversations with the patient. A systematic and customised system for the documentation of end-of-life care could be a means to increase the focus on aspects other than purely physical issues.

## Supplementary Information

**Additional file 1.**

## Data Availability

All data analysed during this study are included in this published article.

## Abbreviation

HCP: Health-care professional
