# Supplementary material for: Documentation of older people’s end-of-life care in the context of specialised palliative care: a retrospective review of patient records
Source: BMC Palliat Care. 2021 Jun 24;20:91. doi: 10.1186/s12904-021-00771-w (PMC8228932; doi:10.1186/s12904-021-00771-w)
Supplement: Supplementary file 1 — Additional file 1. [file 12904_2021_771_MOESM1_ESM.docx]

**Additional file 1.** Documentation of assessment instruments in relation to specific symptoms used by health professionals, (*n*= 345)

| **Symptom** | **Assessment instrument** | **Health care professional** | ***n* (%)** |
| --- | --- | --- | --- |
| Pain | Visual Analog Scale; Numerical Rating Scale  Verbal Descriptive Scale  Abbey Pain Scale; Face, Legs, Activity, Cry, Consolability scale    Integrated Patient care Outcome Scale | Nurse  Physician  Nurse  Physician  Nurse-assistant  Nurse  Nurse | 167 (48)  1 (<1)  88 (26)  1 (<1)  1 (<1)  25 (7)  1 (<1) |
| Anxiety | Visual Analog Scale; Numerical Rating Scale  Face, Legs, Activity, Cry, Consolability scale; Abbey Pain Scale  Verbal Descriptive Scale | Nurse  Nurse  Nurse | 14 (4)  10 (3)  7 (2) |
| Breath/bleeding/  circulation problem | Visual Analog Scale; Numerical Rating Scale  Face, Legs, Activity, Cry, Consolability scale; Abbey Pain Scale    Norton Pressure Sore Risk-Assessment Scale | Nurse  Nurse  Nurse | 5 (1.5)  1 (<1)  1 (<1) |
| Nutrition problem | Revised Oral Assessment Guide  Visual Analog Scale; Numerical Rating Scale  Integrated Patient care Outcome Scale | Nurse  Nurse  Nurse | 1 (<1)  5 (1.5)  3 (1) |
| Unresponsiveness,  life last time | Visual Analog Scale; Numerical Rating Scale  Face, Legs, Activity, Cry, Consolability scale; Abbey Pain Scale | Nurse  Nurse | 5 (2)  3 (1) |
| Tiredness | Visual Analog Scale; Numerical Rating Scale  Verbal Descriptive Scale  Face, Legs, Activity, Cry, Consolability scale; Abbey Pain Scale | Nurse  Nurse  Nurse  Physician | 1 (<1)  1 (<1)  1 (<1)  1 (<1) |
| Fall/fall tendency | Downton Fall Risk Index  Visual Analog Scale; Numerical Rating Scale | Nurse  Nurse | 1 (<1)  1 (<1) |
